# Supplementary material for: Self-Limitations of Heat Release in Coupled Core-Shell Spinel Ferrite Nanoparticles: Frequency, Time, and Temperature Dependencies
Source: Nanomaterials (Basel). 2021 Oct 26;11(11):2848. doi: 10.3390/nano11112848 (PMC8624666; doi:10.3390/nano11112848)
Supplement: Supplementary file 1 [file nanomaterials-11-02848-s001.zip › nanomaterials-1423085-supplementary.pdf]

Supplementary Materials

Self-Limitations of Heat Release in Coupled Core-Shell Spinel Ferrite Nanoparticles: Frequency, Time, and Temperature Dependencies

Shankar Khanal [1], Marco Sanna Angotzi [2,3], Valentina Mameli [2,3], Miroslav Veverka [1], Huolin L. Xin [4], Carla Cannas [2,*] and Jana Vejpravová [1,*]

1 Department of Condensed Matter Physics, Faculty of Mathematics and Physics, Charles University, Ke Karlovu 5, 121 16 Prague 2, Czech Republic; skhanal@mag.mff.cuni.cz (S.K.); miroslavveverkag@gmail.com (M.V.)
2 Department of Chemical and Geological Sciences, University of Cagliari, S.S. 554 bivio per Sestu, 09,042 Monserrato (CA), Italy; sannamarco91@gmail.com (M.S.A.); valentina.mameli@unica.it (V.M.)
3 Consorzio Interuniversitario Nazionale per la Scienza e Tecnologia dei Materiali (INSTM), Via Giuseppe Giusti 9, 50,121 Firenze (FI), Italy
4 Department of Physics and Astronomy, University of California, Irvine, CA 92617, USA; huolinx@uci.edu
* Correspondence: ccannas@unica.it (C.C.); jana@mag.mff.cuni.cz (J.V.)

I. Structural, static, and dynamic magnetic properties

Table S1. Selected parameters of the MNPs: unit cell parameter (a), the crystallite size (D_{XRD}) obtained by the Rietveld analysis, volume-weighted particle size obtained from the TEM analysis using n particles (D_{TEM}), shell thickness (Δ_{TEM}), and magnetic diameter (D_{MAG}) derived from the median magnetic moment, μ_m (Table S2).

Sample	*a*(Å)	D_{XRD}# (nm)	n	D_{TEM} (nm)	SD (TEM)	Δ_{TEM} (nm)	D_{MAG}# (nm)	D_H (nm)
Co1	8.38(1)	6.3 ± 0.6	8300	7.5	1.1	-	5.1 ± 0.5	29 ± 6*
Co2	8.38(1)	7.1 ± 0.7	3622	9.0	1.3	-	5.3 ± 0.5	31 ± 6
Co1@Fe	8.35(1)	8.8 ± 0.9	2885	12.8	1.7	2.7	6.5 ± 0.7	34 ± 7
Co1@Mn	8.41(1)	8.6 ± 0.9	2553	13.2	1.6	2.9	6.3 ± 0.6	35 ± 7
Co2@Fe	8.36(9)	10.2 ± 1.0	6666	11.7	1.5	1.4	8.4 ± 0.8	33 ± 7**
Co2@Mn	8.44(9)	8.8 ± 0.9	3889	13.3	1.7	2.1	6.9 ± 0.7	35 ± 7

*value based on the ref. [2] **experimental value #the minimum uncertainties are estimated as 10%; both the Rietveld analysis and analysis of the log-normal distribution of the magnetic moments underestimate the uncertainty in determination of the absolute values: Rietveld – in order of 0.01 nm, log-normal distribution of the μ ~0.1 nm.

Table S2. Magnetic parameters of the cores and core-shell MNPs adopted from our previous work [1]. T_b – blocking temperature, $T_{b,\,diff}$ – furcation temperature of the ZFC-FC curves, μ_m – mean magnetic moment and σ – standard deviation of the log-normal distribution f(μ), M_s at 10 K/300 K - saturation magnetization. The characteristic Néel, Brown and resulting (effective) relaxation times are also given. The τ_b values are based on the hydrodynamic diameters of the MNPs given in Table S1. The last two columns show the τ_r values for the lower and upper bound of the τ_b based on the D_H = 20 nm and 40 nm.

Sample	T_b (K)	$T_{b,\,diff}$ (K)	μ_m (μ_B)	σ	τ_n (s)	τ_b (s)	τ_r (s)	M_s (Am²/kg)	τ_r (20 nm) (s)	τ_r (40 nm) (s)
Co1	163	266	3.9×10^3	0.93	6×10^{-7}	1.6×10^{-5}	5.8×10^{-7}	90/74	5.4×10^{-7}	5.9×10^{-7}
Co2	206	313	4.4×10^3	0.95	4×10^{-6}	1.9×10^{-5}	3.3×10^{-6}	92/77	2.2×10^{-6}	3.6×10^{-6}
Co1@Fe	237	333	6.5×10^3	0.48	5×10^{-3}	2.5×10^{-5}	2.5×10^{-5}	94/81	5.1×10^{-6}	4.1×10^{-5}
Co1@Mn	233	312	6.2×10^3	0.69	7×10^{-4}	2.7×10^{-5}	2.6×10^{-5}	94/75	5.1×10^{-6}	3.9×10^{-5}
Co2@Fe	190	270	15.8×10^3	0.30	7×10^{-6}	2.3×10^{-5}	5.4×10^{-6}	89/77	3.0×10^{-6}	6.0×10^{-6}
Co2@Mn	216	295	10.8×10^3	0.52	5×10^{-5}	2.7×10^{-5}	1.8×10^{-5}	92/70	4.6×10^{-6}	2.3×10^{-5}

Table S3. First-order anisotropy constants, *K* calculated using the eq. S6, adopted from [1] and critical diameters of the monodomain state, d_c and $d_{c,\,diff}$ at T_b and $T_{b,\,diff}$, respectively, using eq. S7.

Sample	*K* (10^4 J/m^3)	d_c (nm)	$d_{c,\,diff}$ (nm)
Co1	75	5.0	6.0
Co2	89	5.2	6.0
Co1@Fe	56	6.4	7.1
Co1@Mn	44	6.8	7.5
Co2@Fe	31	7.2	8.1
Co2@Mn	30	7.6	8.4

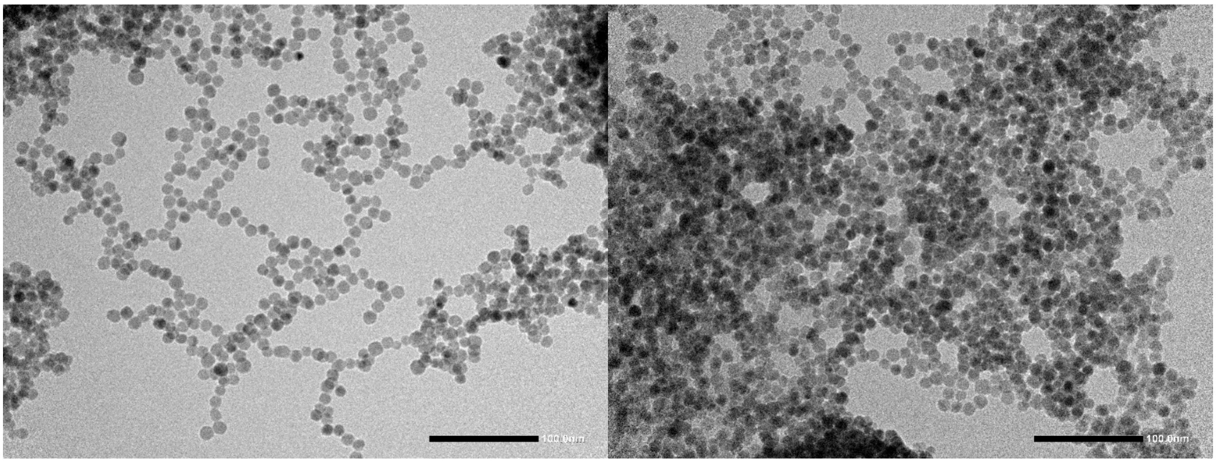

Figure S1. TEM micrographs of oleate-capped Co1@Mn sample (left) and Co1@Mn intercalated with CTAB (right).

II. Important relations used for evaluation of the magnetic parameters

For the real system of the superparamagnetic MNPs with a size distribution, the magnetization, *M* in the magnetic field, *H* can be written as a weighted sum of the Langevin functions:

$$(H,T) = \int_0^\infty \mu L\left(\frac{\mu H}{k_B T}\right) f(\mu) d\mu + \chi_{linear} H \quad \text{(S1)}$$

where $f(\mu)$ corresponds to the unimodal log-normal distribution of the magnetic moments, μ expressed as:

$$f(\mu) = \frac{1}{\sqrt{2\pi}\mu\sigma} exp\left(-\frac{ln^2\left(\frac{\mu}{\mu_0}\right)}{2\sigma^2}\right), \mu_m = \mu_0 exp\frac{\sigma^2}{2} \quad \text{(S2)}$$

where σ is the distribution width, μ_0 and μ_m are the median and mean magnetic moment, respectively. The second term in the equation (S1) corresponds to an additional linear contribution to the magnetization, originating from diamagnetic or paramagnetic components of the sample (usually from the disordered parts of MNPs). The parameters of $f(\mu)$ were obtained from the refinement of the magnetization isotherm measured above T_b in Matlab using equation (Eq. S1).

The median magnetic size, D_{MAG} of the particle was calculated from the μ_m using the expression:

$$D_{\text{MAG}} = \sqrt[3]{\frac{6\mu_0 a^3}{\mu_{uc}\pi}}, \quad \text{(S3)}$$

where *a* and μ_{uc} are the lattice parameter and the magnetic moment of the unit cell of the spinel phase, respectively.

The ZFC curves were fitted in Matlab [4]by the least squares' method implemented in our own-written script using the following formula:

$$M_{ZFC}(T) \propto C + A\left[\frac{25}{t}\int_0^t t_B f(t_B)dt_B + \int_t^{\infty} f(t_B)dt_B\right] \quad (S4)$$

where the C is a correction to the random orientation of the freezing moments resulting in a random nonzero value of the low-temperature magnetization and the A is a fraction-scaling constant, which is refined together with the T_b and σ as the exact value of the M_S and K are usually not known.

The distribution of the blocking temperatures f (T_b) was also estimated using a simple empirical relation:

$$f(T_b) \propto \frac{-d[M_{FC}(T) - M_{ZFC}(T)]}{dT} \quad (S5)$$

The K values were determined using the T_b and D_{MAG} as follows:

$$K = \frac{25k_BT_b}{V} \quad (S6)$$

where k_B corresponds to the Boltzmann constant.

The critical diameter of the monodomain, d_c at a given temperature, corresponding to the blocking temperature can be estimated using the following relation [5]:

$$d_c = \sqrt[3]{V_c};\ V_c = \frac{23k_BT}{K}. \quad (S7)$$

III. Heating properties – additional data and calculations

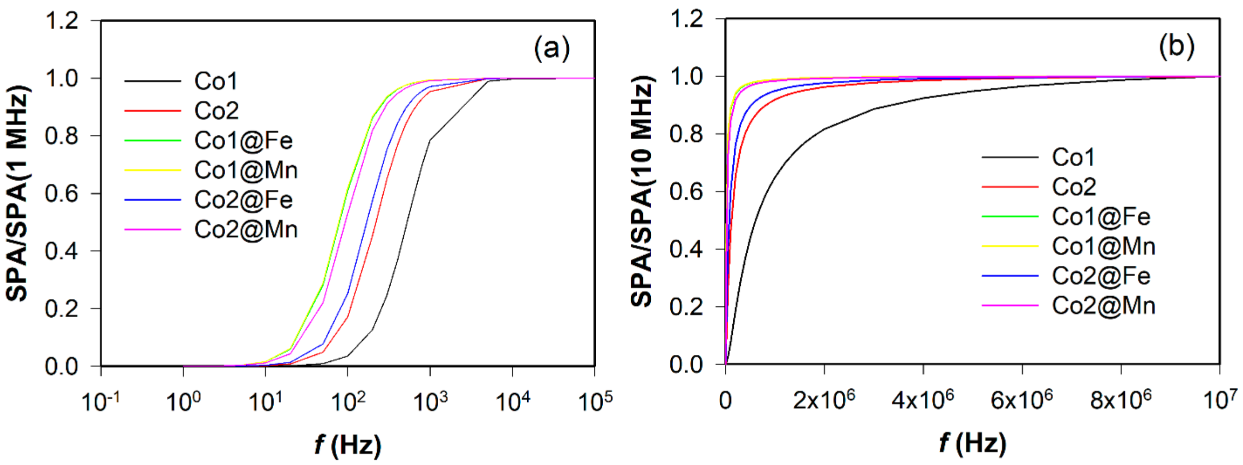

Figure S2. Calculated SPA/SPA_{max} vs. frequency dependence based on the Equation 8 (**a**) and Equation 6 (**b**), normalized to the SPA at 1 MHz and 10 MHz, respectively.

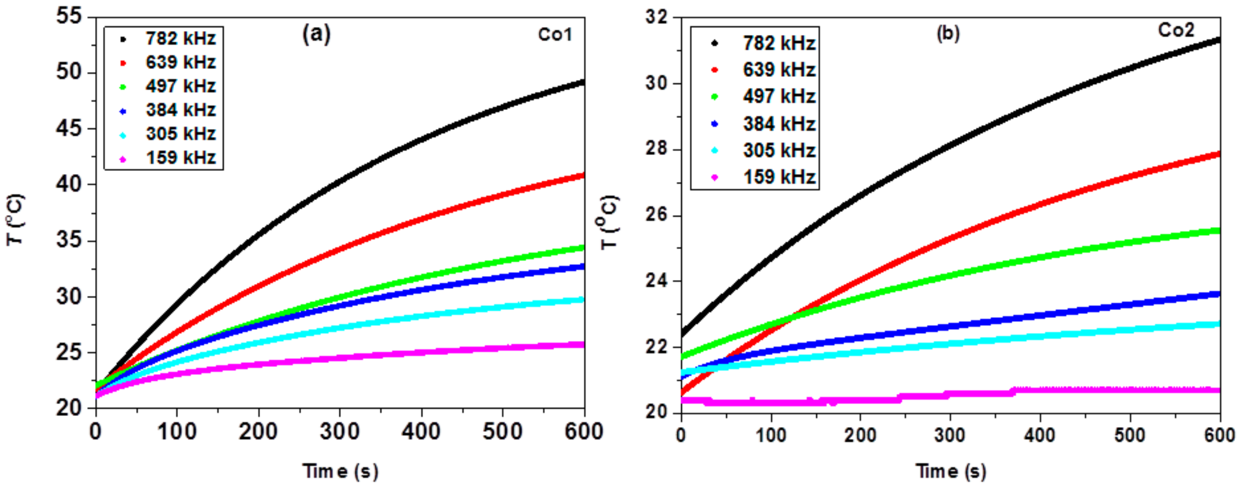

Figure S3. Heating curves at various frequencies with amplitude of AMF 31.6 mT for the Co1 (**a**) and Co2 (**b**) samples.

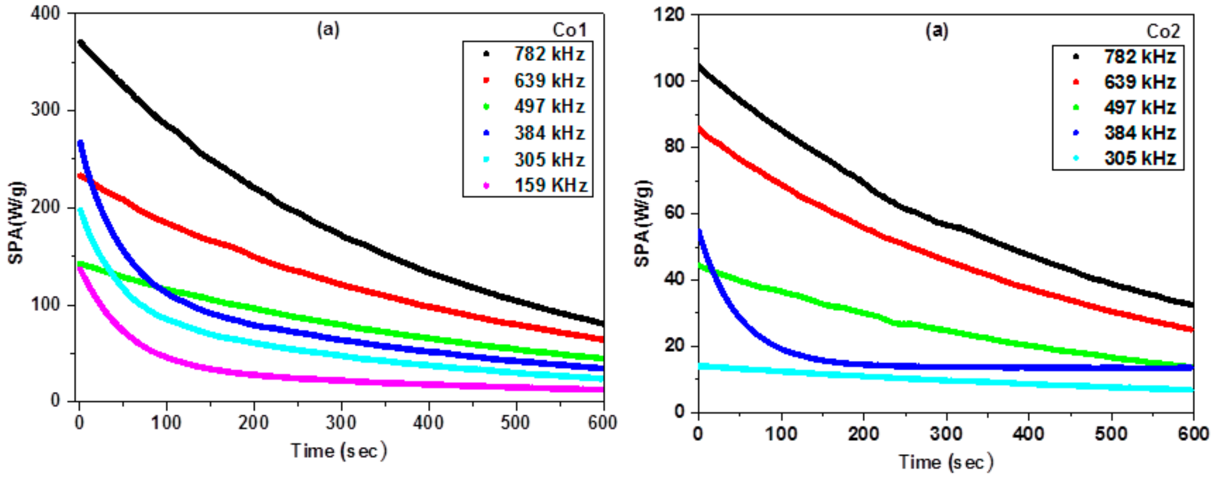

Figure S4. SPA vs. time evaluated at various frequencies with amplitude of AMF 31.6 mT for the Co1 (**a**) and Co2 (**b**) samples.

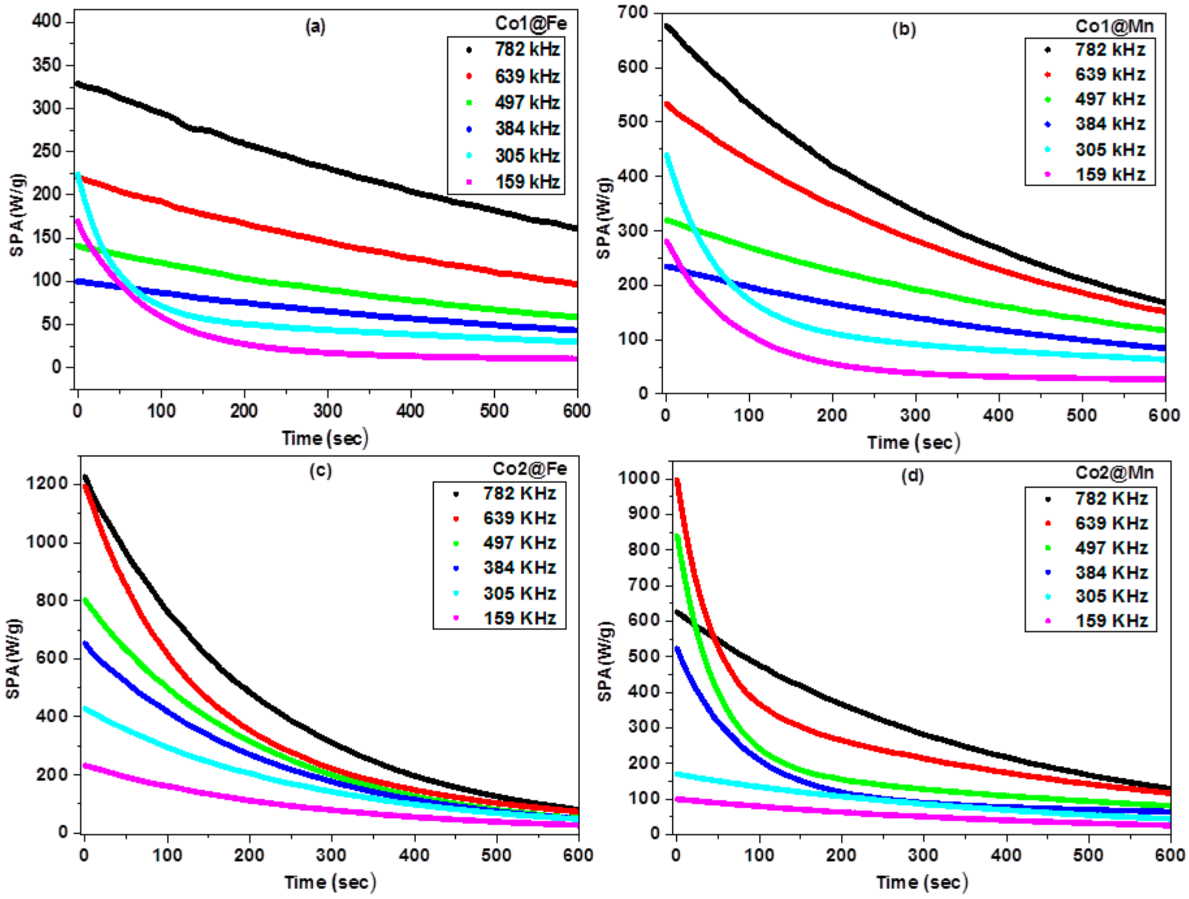

Figure S5. SPA vs. time evaluated at various frequencies with amplitude of AMF 31.6 mT for Co1@Fe, Co1@Mn, Co2@Fe, and Co2@Mn **a**–**d**.

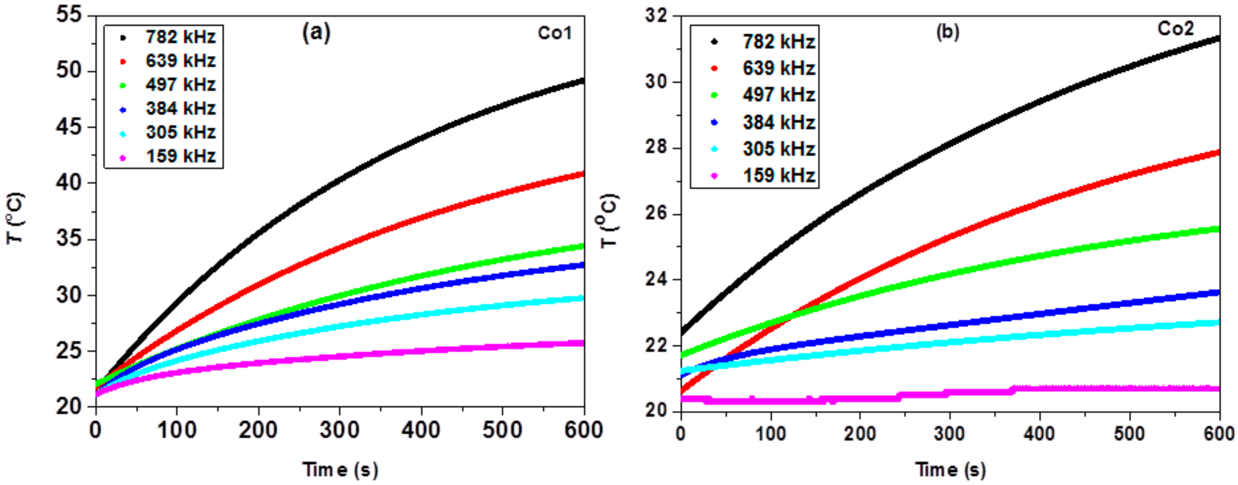

Figure S6. SPA vs. temperature evaluated at various frequencies with amplitude of AMF 31.6 mT for Co1 (**a**) and Co2 (**b**).

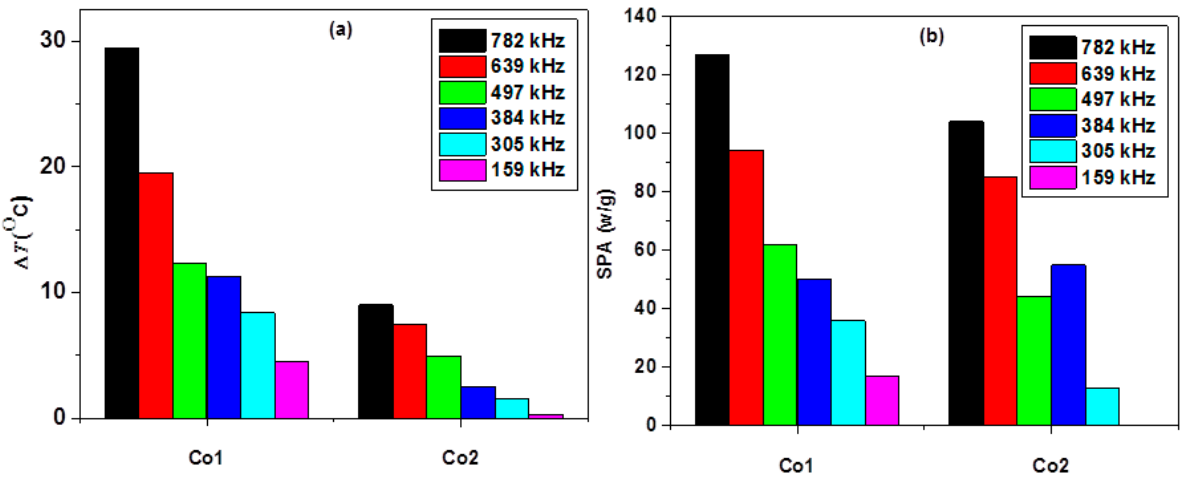

Figure S7. Frequency dependence of the ΔT (**a**) and SPA (**b**) for Co1 and Co2 samples.

Table S4. SPA (W/g)/ΔT (°C) evaluated at the initial phase for f = 305, 497, and 782 kHz and identical amplitude of 31.6 mT.

Sample	305 kHz	497 kHz	782 kHz
Co1@Fe	224/8.7	141/15.6	327/36.1
Co1@Mn	439/17.5	315/29.2	746/54.6
Co2@Fe	428/25.1	800/41.1	1223/61.2
Co2@Mn	416/13.2	840/25.8	626/41.7

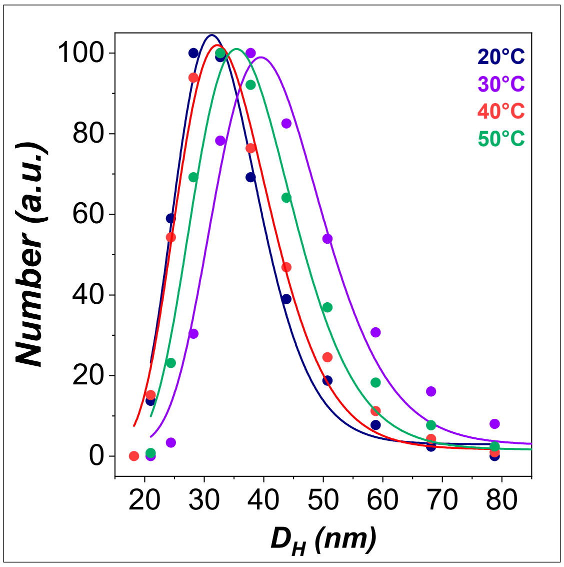

Figure S8. Temperature dependence of the $_{\mathrm{H}}$ for Co2@Fe sample.

Table S5. D_{H}, SD and τ_b values at selected temperatures obtained for the Co2@Fe sample.

T (°C)	D_{H} (nm)	SD (nm)	τ_b (s)
20	32.8	7.6	$1(1)\cdot10^{-5}$
30	41.6	9.9	$2(1)\cdot10^{-5}$
40	30.1	8.5	$7(2)\cdot10^{-6}$
50	37.4	9.2	$1(1)\cdot10^{-5}$

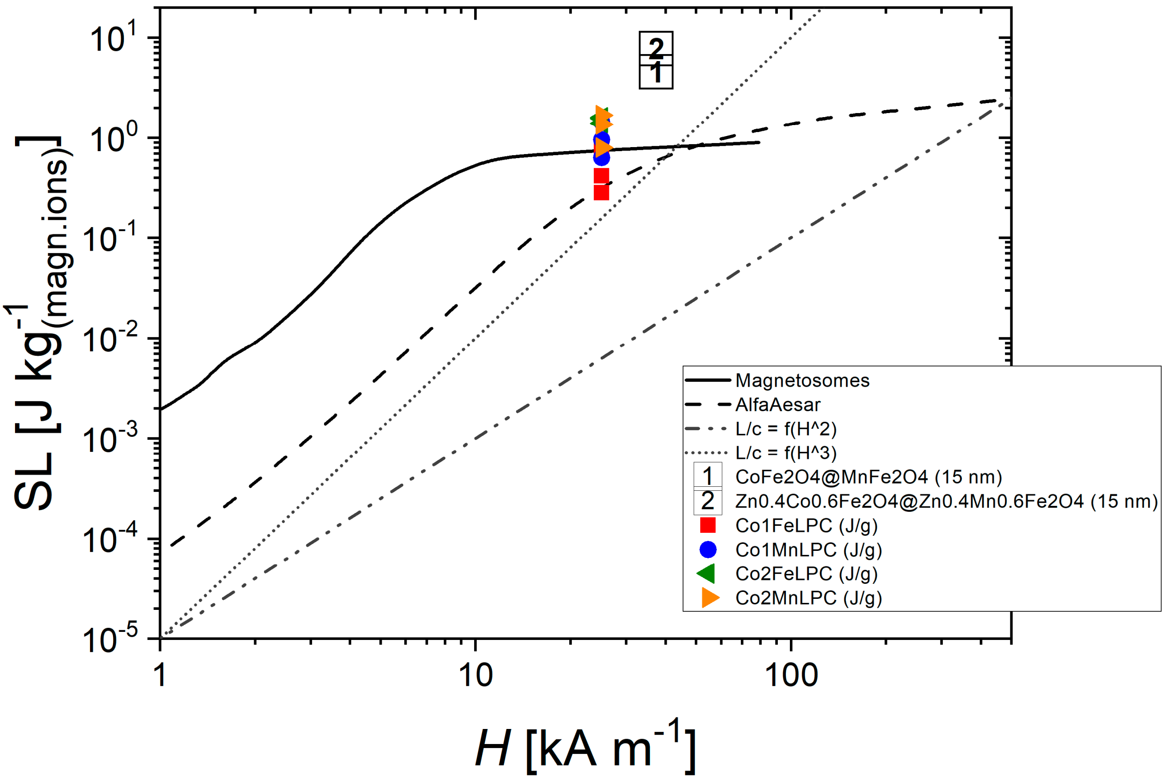

Figure S9. Comparison of the heating efficiency per one cycle (S.L. = SPA/f) for various types of MNPs and the core-shell samples studied in this work.

References

1. Van Rijssel, J.; Kuipers, B.W.M.; Erné, B.H. Non-regularized inversion method from light scattering applied to ferrofluid magnetization curves for magnetic size distribution analysis. *J. Magn. Magn. Mater.* **2014**, *353*, 110–115, doi:10.1016/j.jmmm.2013.10.025.
2. Mameli, V.; Musinu, A.; Ardu, A.; Ennas, G.; Peddis, D.; Niznansky, D.; Sangregorio, C.; Innocenti, C.; Thanh, N.T.K.; Cannas, C. Studying the effect of Zn-substitution on the magnetic and hyperthermic properties of cobalt ferrite nanoparticles. *Nanoscale* **2016**, *8*, 10124–10137, doi:10.1039/c6nr01303a.
3. Repko, A.; Nižňanský, D.; Poltierová-Vejpravová, J. A study of oleic acid-based hydrothermal preparation of CoFe2O4 nanoparticles. *J. Nanoparticle Res.* **2011**, *13*, doi:10.1007/s11051-011-0483-z.
4. MATLAB *Win64 (R2020a)*; The MathWorks Inc.: Natick, Massachusetts, 2010.
5. Knobel, M.; Nunes, W. C.; Socolovsky, L. M.; De Biasi, E.; Vargas, J. M.; Denardin, J. C. Superparamagnetism and Other Magnetic Features in Granular Materials: A Review on Ideal and Real Systems. *Journal of Nanoscience and Nanotechnology*. **2008**, 8, 2836-2857. https://doi.org/10.1166/jnn.2008.15348
